# Questionnaire development and validity to measure sexual intention among youth in Malaysia

**DOI:** 10.1186/s12889-016-3949-1

**Published:** 2017-02-02

**Authors:** Noor Azimah Muhammad, Khadijah Shamsuddin, Rahmah Mohd Amin, Khairani Omar, Ramayah Thurasamy

**Affiliations:** 1 Department of Family Medicine, Faculty of Medicine, Universiti Kebangsaan Malaysia, Jalan Yaacob Latiff, 56000 Cheras, Kuala Lumpur Malaysia; 2 Department of Community Health, Faculty of Medicine, Universiti Kebangsaan Malaysia, Jalan Yaacob Latiff, 56000 Cheras, Kuala Lumpur Malaysia; 3 Faculty of Medicine and Health Sciences, Universiti Sultan Zainal Abidin, Medical Campus, Jalan Sultan Mahmud, 20400 Kuala Terengganu, Terengganu Malaysia; 4 Faculty of Medicine and Health Sciences, Universiti Sains Islam Malaysia, Bandar Baru Nilai, 71800 Nilai, Negeri Sembilan Malaysia; 5 School of Management, Universiti Sains Malaysia, 11800 Minden, Pulau Pinang Malaysia

**Keywords:** Adolescent, Attitude, Confirmatory factor analysis, Exploratory factor analysis, Self-efficacy, Sexual intention, Social norms, Questionnaire

## Abstract

**Background:**

From the Theory of Planned Behaviour perspective, sexual intention is determined by a permissive attitude, perception of social norms and perceived self-efficacy in performing sexual activity. The aim of this study was to develop and validate the Youth Sexual Intention Questionnaire (YSI-Q), which was designed to measure sexual intention among youths in Malaysia.

**Methods:**

A total of 25 items were developed based on literature reviews encompassing four main constructs: sexual intention, attitude, social norms and self-efficacy. The YSI-Q then underwent a validation process that included content and face validity, exploratory factor analysis (EFA), reliability analysis, and confirmatory factor analysis (CFA). This study was conducted on unmarried youths aged 18 to 22 years who were studying in colleges around Klang Valley, Malaysia.

**Results:**

EFA supported the four factor structure, but five items were removed due to incorrect placement or low factor loading (<0.60). Internal reliability using Cronbach’s alpha ranged between 0.89 and 0.94. The CFA further confirmed the construct, convergent and discriminant validity of the YSI-Q with *χ* ^2^ = 392.43, df = 164, *p* < 0.001, *χ* ^2^/df = 2.40, CFI = 0.93 and TLI = 0.92 and RMSEA = 0.08.

**Conclusion:**

The final set of YSI-Q consisted of 20 items measuring sexual intention (five items), attitude (five items), social norms (six items) and self-efficacy (four items) of practicing sexual activity. YSI-Q was shown to be a reliable and valid tool to be used among Malaysian youths.

**Electronic supplementary material:**

The online version of this article (doi:10.1186/s12889-016-3949-1) contains supplementary material, which is available to authorized users.

## Background

Sexual activity among youths is a global phenomenon affecting about 30 to 50% of young men and women from developed and African countries, where the peak age for sexual initiation is between 15 and 19 years of age [1–4]. A lower prevalence of about 5 to 13% was reported in Asian countries such as Malaysia [5, 6] but is expected to be on the rise due to the globalisation of mass media, delayed marriage and improvement in social status. In general, youths especially from developing countries including Malaysia, are practicing unsafe sexual activities such as having sex with multiple partners and sex without condom or contraception. Only about a third of sexually active youths in China and Malaysia used condoms every time they had sex [7, 8]. Hence, the unsafe sexual activities predispose them to detrimental complications that include sexually transmitted diseases including Human Immunodeficiency Virus (HIV) infection, unplanned pregnancy, illegal abortions and baby dumping. Worldwide, about 2 million youths aged 10–19 years were infected by the HIV virus and 20% of girls below 18 years were pregnant [9].

Malaysia, as many other Asian countries, is a conservative country that does not allow sexual activity among youths except those who are legally married. The legal age of marriage is 18 years old and on average, Malaysians get married at 26 to 29 years old once they have completed their studies in college. Nevertheless, there are Malaysian youths who are sexually active and practice premarital sex despite the social restriction. In order to understand the complex issues related to youth sexual activity, improving the understanding of youth sexual intention is necessary. Studies have shown that changing behavioural intention may lead to a positive behavioural outcome [10, 11], and this logic may be applied to reduce the range of social, developmental, and physical problems that are associated with youth sexual activity.

The Theory of Planned Behaviour posits that individual behaviour is determined by his or her personal intention on performing a particular behaviour. In the presence of a high intention, a person is motivated to actually execute the behaviour [12]. The three main factors that determine intention are (1) perceived attitude on performing the behaviour (2) perceived social norms and (3) perceived behavioural control over or self-efficacy in performing the behaviour [13].

Attitude is about a person’s belief in performing the behaviour. It has a positive relationship with the actual performance of the behaviour, especially when one foresees the benefits associated with it [10]. Youths with permissive attitudes towards sexual activity are likely to have the intention to perform sexual behaviours [14]. A non-permissive attitude towards premarital sex is common among conservative cultures like the people in Malaysia, Hong Kong and China. However, modernisation may have inadvertently changed people’s attitudes and a permissive attitude is now common among sexually active youths in these countries [15–17].

Social norms cover perceived social pressure on performing the behaviour, approval or disapproval from others, and what is being practiced by significant others [10]. In the context of sexual activity, perceived social norms may include societal pressure to practice premarital sex, approval from significant others such as parents, and common sexual activity practiced or beliefs held by their peers. Youths who perceive that their partner would agree to engage in premarital sex are likely to be associated with having intention to perform sexual activity [14]. Similarly, youths who perceive that premarital sex is allowed by their parents or being practiced by their peers are likely to have the intention to perform premarital sex [18, 19].

Self-efficacy means having the belief in one’s ability to perform a particular behaviour. Self-efficacy depends on past experiences, available resources and anticipated obstacles. Cognitive assessment of environmental cues usually occurs prior to the enactment of any behaviour [12]. Youths are less likely to have sexual intention if they think having sex is difficult and if they have no sexual partner [10]. Their belief in their ability increases with repeated experience in performing the sexual activity [20] and reduces with paucity of resources such as a suitable time and place [21]. A high level of perceived self-efficacy predicts sexual activity [22].

**Fig. 1:**
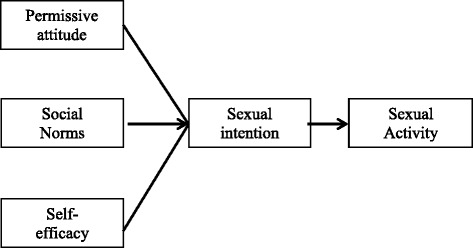
Theoretical framework for sexual activity based on the Theory of Planned Behaviour

In short, based on the Theory of Planned Behaviour, having a permissive attitude towards sexual activity, a perceived presence of social norms together with a high belief in the ability to control or perform the sexual activity will lead to a high intention in performing the sexual activity. Intention mediates the relationship between the cognitive process (attitudes, social norms, self-efficacy) and a behaviour [12]. In our context, the behaviour is sexual activity (Fig. 1).

In addition, human behaviour, including sexual activity, is culturally bound. Thus, the items to measure attitude, perception of social norms, perception of sexual activity and sexual intention of Malaysian youths or those from conservative countries may not be similar to that of youths from western or developed countries. Unfortunately, there is no available questionnaire to measure all the constructs of sexual intention for conservative Asians generally or Malaysians specifically. Thus, the aim of this study was to develop and validate a set of questionnaire to measure sexual intention among Malaysian youths.

## Method

### Questionnaire development

The final set of Youth Sexual Intention Questionnaire (YSI-Q) consists of 20 items as listed in Table 2. The initial set of YSI-Q had 25 items constructed for Youth Sexual Intention (YSI-Q) and were based on literature reviews, expert opinions and in-depth discussions with youths. Using the Theory of Planned Behaviour as the framework (refer Fig. 1), the YSI-Q was developed to measure four constructs: permissive attitude towards sexual activity, perceived social norms, perceived self-efficacy and sexual intention. All of the constructs reflected the same behaviour which was premarital sexual activity [13]. In order to cater to local use, we needed to develop a set of questionnaire in the national language, which is Malay. As the initial version of YSI-Q was in English, the set of questionnaire underwent the standard translation process. Two forward translations into Malay were done by a linguistic expert and a medical doctor. Subsequently, the two sets of translated Malay questionnaires underwent a backward translation into English by another linguistic expert and a medical doctor. The aim of this procedure was to identify translated items that closely match the original English version and to produce the Malay version of the questionnaire [23].

The introductory statement for the set of questions was *“the following statements are about sexual activities among unmarried youths*”. Respondents were requested to give their answers for each item using a 4-point Likert-type scale of 1 (strongly disagree), 2 (disagree), 3 (agree) and 4 (strongly agree). The midpoint neutral answer was avoided in this questionnaire in order to minimise response bias. Midpoint responses are frequently chosen by respondents because they are indecisive, reluctant to answer, or the question does not apply to them [24]. The four point responses have been shown to be optimal for youths [25]. In this questionnaire, the higher score in each factor indicated a higher permissive attitude, perceived social norms, perceived self-efficacy and sexual intention respectively.

### Validation process

The validity of YSI-Q was assessed using content validity, face validity, exploratory factor analysis (EFA), reliability analysis and confirmatory factor analysis (CFA). EFA is useful for the identification of observed items that will best represent latent constructs [26] while CFA is to confirm and validate the measurement model in terms of construct, convergent and discriminant validity [27].

For content validity, a panel of experts that included an Adolescent Psychiatrist, a Family Medicine Consultant who specialised in adolescent health, a Community Health Physician and a Clinical Psychologist reviewed and revised the items. These content experts were asked to assess the appropriateness of items for the respective constructs [28]. Face validity was used to review grammar, syntax, organisation and appropriateness of the questionnaire [28]. This was done by three college students, two females and one male, on separate occasions. These students were different from the group of students who were involved in subsequent analyses. They were requested to give their opinion on the meaning of the items and to suggest suitable phrases to match their age specific lingo if there was any problem with the items.

Data for subsequent factor analysis was extracted from a larger study (*N* = 1026) on Youth Sexual Intention and Activity among college students in Malaysia. The students were randomly selected from twelve colleges in Klang Valley, Malaysia and aged between 18 and 22 years. Married students and students who did not give their consent were excluded from this study. From the data set that was entered into the Statistical Package for the Social Sciences (SPSS) software, 150 samples were randomly selected for exploratory factor analysis (EFA) and another 200 samples for confirmatory factor analysis (CFA). The two groups of samples were independent, whereby 150 samples were selected first and followed by the selection of the 200 samples from the remaining 876 samples. These sample sizes met the minimum requirement of five samples per item for the EFA [27] and of sufficient size for the CFA with a theoretical model containing four constructs [29].

Exploratory factor analysis (EFA) was performed on the first independent 150 samples. Using SPSS, factor analysis began with a decision on the number of factors to be extracted based on an eigenvalue of more than 1, scree plot and parallel analysis. The use of multiple criteria, including parallel analysis, was suggested as an optimal approach in determining the number of factors for extraction [27]. Subsequently, factor analysis was run using principal axis factoring with promax rotation. Items with a factor loading of less than 0.6 or incorrectly placed based on their theoretical meaning were removed from the questionnaire [26]. This was followed by the reliability analysis using Cronbach alpha coefficients for each construct that were established prior to the assessment of construct validity [29].

Confirmatory factor analysis (CFA) was the final part of the validation process, using Analysis of Moment Structure (AMOS) to support the measurement model. This was done on the second independent group of 200 samples. A good model fit was indicated by these indices: the ratio of chi-square to degree of freedom (*χ* ^2^/df) < 5.0, root mean square error of approximation (RMSEA) ≤0.08, comparative fit index (CFI) >0.9, Tucker Lewis Index (TLI) >0.9, and *p* >0.05 for the chi-square test [29, 30]. Convergent validity reflects the degree of items in each construct, is interconnected by matching their theoretical connection and was based on three criteria: factor loadings >0.5, average variance extracted (AVE) for each construct >0.5 and composite reliability (CR) >0.7 [29]. An instrument is considered as having good discriminant validity when the items are unrelated theoretically and are indeed unrelated in the measurement model. Discriminant validity was achieved when AVE values for any two constructs were greater than the squared correlations between the two construct [29].

## Results

### Content and face validity

For content validity, all of the experts unanimously agreed with the suggested items and felt that the items were relevant and consistent with the intended constructs. Likewise, from the in-depth interviews with the three students, they found the items to be relevant and appropriate for their age. Overall, they had no difficulty in understanding the items. However, there were three items that were amended based on the feedback from the students, in order to improve the clarity of the questions. Item ‘*I believe one-night stands are all right*’ was changed to ‘*I believe a sexual encounter that lasts only once is all right*’, item ‘*to have sex at my age is a norm*’ was changed to ‘*to have sex before marriage is a norm for a youth like me*’ and item ‘*I feel under social pressure to have sex at my age*’ was changed to ‘*I feel there is pressure from the people surrounding me to have sex at my age*’. The final set of YSI-Q at this stage contained twenty-five items.

### Descriptive statistics for EFA and CFA samples

The sample for EFA and CFA was extracted from a sample of 1,026 college students. More than half of them were from urban areas (66.6%), came from low to middle income families with a monthly income of less than RM5000 (70.4%) and stayed out of their homes in places such as hostels or with friends (65.0%). Most of their parents had a low level of education with only 30.6% having a college or university education. There were 63.4% Muslims, 21.2% Buddhists, 7.5% Hindus, 5.8% Christians, and 2.2% with other religions (Atheist, Bahais or Taoism) or no religion. The mean age of the respondents was 19.8 (SD = 1.3) years with more than half (58.7–59.0%) being females in both the EFA and CFA groups. There were no significant differences in the age and gender between the two groups. Malays formed the majority (58.0–62.0%) in both groups, followed by Chinese (30.5–30.7%). There was a higher proportion of Indians in the EFA group (11.3%) compared to the CFA group (4.5%), *p* < 0.02.

### Exploratory factor analysis

The Kaiser-Meyer-Olkin (KMO) measures the confirmed sampling adequacy, KMO = 0.90. The Barlett’s Test of Sphericity was significant, *p* < 0.01 and supported the factorability of the items. From the initial analysis, based on scree plot and the Kaiser-Guttman criterion of eigenvalues of more than 1, there were four factors to be extracted. This was further supported by the parallel analysis using the Monte Carlo simulation technique. Thus, four-factor extraction was decided as the most statistically and conceptually suitable and these four factors explained 70.0% of the variance.

Table 1 shows the factor loadings before (25 items) and after (20 items) following the removal of five items in YSI-Q. From the first exploratory factor analysis (25 items), four items from social norms were incorrectly placed into the attitude construct and one item of self-efficacy showed a factor loading of less than 0.6. The social norms items were one item for perceived community norms: ‘*I feel there is pressure from the people surrounding me to have sex at my age*’ and three items for perceived parental norms ‘*My parents allow me to have sex at my age now’*, ‘*My parents allow me to have sex with my partner*’ and ‘*My parents allow me to have sex provided I know how to avoid pregnancy*’. The item for self-efficacy was ‘*For me, to have sex is easy’.* Hence, these five items were removed from the YSI-Q and a cleaner result was obtained with a factor loading of or more than 0.6 (see Table 1).

**Table 1:** Four factor loadings of the YSI-Q 25 items and YSI-Q 20 items

	25 items					20 items				
YSI-Q item	Factor loadings					Factor loadings				
	SI	AT	SN	SE	Communality	SI	AT	SN	SE	Communality
SIa	**.85**	.12	.16	.21	0.80	**.85**	.12	.16	.21	0.81
SIb	**.93**	.13	.12	.28	0.97	**.94**	.14	.18	.27	0.98
SIc	**.88**	.14	.18	.24	0.88	**.89**	.13	.18	.24	0.90
SId	**.67**	.36	.19	.30	0.71	**.66**	.37	.19	.29	0.69
SIe	**.64**	.44	.28	.18	0.71	**.62**	.48	.27	.17	0.72
ATa	.32	**.62**	.25	.06	0.56	.28	**.73**	.25	.06	0.68
ATb	.05	**.57**	.37	.04	0.47	.01	**.63**	.38	.04	0.54
ATc	.17	**.80**	.24	.09	0.73	.15	**.78**	.29	.09	0.72
ATd	.27	**.68**	.17	.27	0.64	.23	**.74**	.18	.28	0.71
ATe	.15	**.62**	.32	.01	0.51	.14	**.59**	.34	.00	0.49
SNC	.28	.43	.40	.18	0.45	–	–	–	–	–
SNPa	.13	.84	.28	.13	0.82	–	–	–	–	–
SNPb	.08	.89	.22	.05	0.85	–	–	–	–	–
SNPc	.08	.90	.23	.09	0.87	–	–	–	–	–
SNFa	.16	.19	**.72**	.23	0.62	.17	.13	**.74**	.22	0.64
SNFb	.19	.28	**.75**	.14	0.69	.19	.25	**.76**	.14	0.70
SNFc	.14	.39	**.73**	.03	0.71	.15	.33	**.76**	.02	0.72
SNFd	.11	.24	**.80**	.15	0.74	.11	.21	**.81**	.16	0.74
SNFe	.17	.34	**.76**	.20	0.76	.18	.30	**.77**	.19	0.75
SNFf	.09	.39	**.72**	.15	0.70	.08	.38	**.73**	.15	0.70
SEa	.24	.17	.42	.45	0.47	–	–	–	–	–
SEb	.22	.01	.22	**.76**	0.67	.23	.01	.22	**.73**	0.63
SEc	.24	.05	.16	**.84**	0.79	.25	.05	.16	**.81**	0.76
SEd	.25	.11	.08	**.83**	0.76	.25	.13	.09	**.85**	0.81
SEe	.13	.19	.11	**.77**	0.66	.12	.19	.14	**.79**	0.70

*SI* sexual intention, *AT* attitude, *SNC* social norms community, *SNP* social norms parents, *SNF* social norms friends, *SE* self-efficacy

The highest values of factor loadings are in bold

At this stage, the YSI-Q contained 20 items, having five items for sexual intention and attitude, six items for social norms and four items for self-efficacy. This explained 71.7% of the variance in which social norms contributed 44.5%, intention contributed 13.5%, attitude contributed 8.1% and self-efficacy contributed 5.6% of the variance. The communality values of the 20 items were at or above 0.5, indicating that each item fitted well with other items in its construct [31].

### Internal reliability

The overall Cronbach’s alpha value for all 20 items was 0.93. The reliability analysis for the sexual intention construct was 0.93, attitude construct was 0.89, social norms construct was 0.94 and self-efficacy construct was 0.90. All values were higher than 0.7 [26], suggesting adequate internal reliability (see Table 2).

**Table 2:** Internal reliability and convergent validity of YSI-Q 20 items

Constructs	Internal reliability	Factor loading	AVE	CR
Sexual intention				
a. I expect to have sex with my partner	0.93	0.95	0.730	0.930
b. I want to have sex with my partner		0.95		
c. I intend to have sex with my partner		0.95		
d. I would like to have sex to see what it is like.		0.78		
e. I would have sex now if I could find a partner who would do it with me		0.58		
Attitude				
a. I believe a sexual encounter that lasts only once is all right.	0.89	0.79	0.622	0.890
b. I believe youths who have never been involved in sexual intercourse before marriage are old-fashioned.		0.77		
c. Youths should have sex before their marriage to see whether they are suited to each other.		0.83		
d. Youths can have sex provided they use methods to stop pregnancy.		0.86		
e. Youths can have sex if they are unable to control their sexual desire.		0.68		
Social norms				
a. Most of my friends are practicing sex before marriage.	0.94	0.86	0.724	0.940
b. Most of my friends think it is mature to practice sex at my age.		0.81		
c. Most of my friends think female youths do not have to maintain their virginity.		0.79		
d. Most of my friends think that male youths are allowed to practice sex before marriage.		0.90		
e. Most of my friends think that you can have sex before marriage if you are in love.		0.89		
f. Most of my friends think that youths who have never been involved in sexual intercourse before marriage are old-fashioned.		0.85		
Self-efficacy				
a. I know when I can have sex.	0.90	0.72	0.712	0.907
b. I know where I can have sex.		0.91		
c. I can decide on my sexual activity.		0.92		
d. Whether I have sex or not is entirely up to me.		0.81		

### Confirmatory factor analysis

**Fig. 2:**
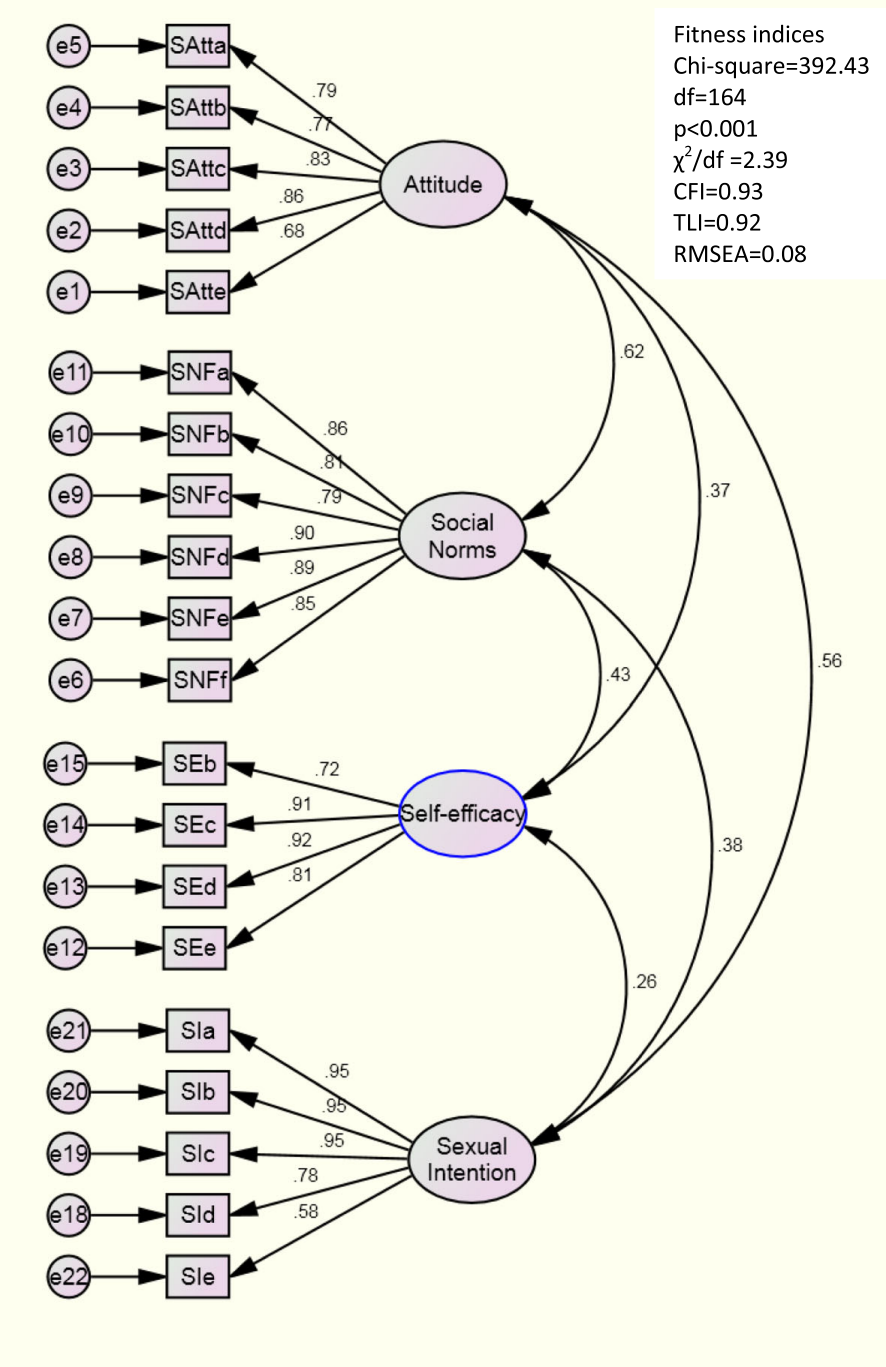
YSI-Q measurement model

Figure 2 illustrates the four-factor model of the 20 items with free correlations among the latent variables. The inter-correlation of constructs ranged from 0.26 to 0.62, indicating no multicollinearity between the constructs. Although the Chi-square test was significant (*χ* ^2^ = 392.43, df = 164, *p* < 0.001), other indices met the recommended criteria for a good model fit: *χ* ^2^/df = 2.40, CFI = 0.93 and TLI = 0.92 and RMSEA = 0.08 [29, 30]. These findings confirmed the construct validity of the YSI-Q measurement model.

Subsequently, YSI-Q was assessed for convergent and discriminant validity. From Table 2, all items and four constructs of YSI-Q met the requirements for convergent validity. The factor loadings ranged from 0.58 to 0.95 and were greater than the recommended level of 0.5. The minimum CR value was 0.89 and exceeded the suggested level of 0.7. AVE varied between 0.62 and 0.73, which was above the recommended level of 0.5 [29]. From Table 3, the square root of AVE for each construct (ranged from 0.79 to 0.85) was more than the correlation coefficient of any pair of the constructs (ranged from 0.31 to 0.59), supporting the discriminant validity of the YSI-Q.

**Table 3:** Discriminant validity of YSI-Q constructs

Constructs	(1)	(2)	(3)	(4)
1. Sexual intention	**0.854**			
2. Attitude	0.593	**0.789**		
3. Social norms	0.430	0.578	**0.851**	
4. Self-efficacy	0.307	0.335	0.424	**0.844**

The square root of AVEs are represented in bold and other values represented correlation

### The final questionnaire

In the final set of questions in the Youth Sexual Intention Questionnaire (YSI-Q), there were 20 items with four constructs, namely (1) Sexual intention, (2) Attitude towards premarital sex (3) Social norms and (4) Self-efficacy. The sexual intention construct consists of five items that reflects current youth intention to perform sexual activity. The Cronbach alpha of sexual intention was 0.93 with factor loadings of each item ranging from 0.58 to 0.95. The attitude towards premarital sex construct refers to having a permissive attitude towards premarital sex and composed of five items. The factor loadings were between 0.68 and 0.79 with a Cronbach alpha of 0.89. The social norms construct comprised of six items that focuses on youth perception of what is being practiced or perceived by their peers. The Cronbach alpha for this scale was 0.94 with factor loadings ranged from 0.79 to 0.90. The self-efficacy construct with a Cronbach alpha of 0.90 refers to youth perception of their ability to perform sexual activity. The factor loadings for the self-efficacy construct ranged from 0.72 to 0.92. The measurement model of YSI-Q with 20 items was found to have good reliability, construct, convergent and discriminant validity.

## Discussion

The primary aim of this study was to develop and validate a set of questionnaire to measure sexual intention among Malaysian youths. Overall, the Youth Sexual Intention Questionnaire (YSI-Q) was successfully developed and validated for Malaysian use.

From the exploratory factor analysis, it was shown that the factorial structure of the initial 25 items corresponded perfectly with the conceptual factors except for five items. This is perhaps partly contributed by the introductory statement: “*the following statements are about sexual activities among unmarried youths*” that appeared before the 25 items. This introductory statement perhaps made the unmarried respondents focus on reflecting their attitude and perception of premarital sex. Specifying the questions in a predictable environment or situation improves the questionnaire construction [12].

Out of the five items that were removed, four items were conceptually designed to represent social norms and one item from the self-efficacy construct. The statement “*I feel there is pressure from the people surrounding me to have sex at my age*” intended to represent the perception of community norms and “My *parents allow me to have sex at my age now*”, “*My parents allow me to have sex with my partner*’ and “*My parents allow me to have sex provided I know how to avoid pregnancy*” representing the perception of parental norms were loaded on a wrong construct. All of these items loaded highly on attitude instead of on social norms. This may reflect the existing conservatism of Asian, and in particular Malaysian, society where there is no pressure from the community and there is no parental approval for premarital sexual activity among youths. Although premarital sex is a global phenomenon, environmental factors may still play their part in shaping youths’ attitude and beliefs towards sexual activity [1]. As such, these constructs carried little weight in the perception of social norms among youths. However, six items that reflect the perception of peer norms such as *“Most of my friends are practicing sex before marriage”, “Most of my friends think that male youths are allowed to practice sex before marriage*” and “*Most of my friends think that youths who have never been involved in sexual intercourse before marriage are old-fashioned*” loaded appropriately on the social norms construct. This is because perception of peers’ sexual activity is stronger than parental or other people’s norms in representing social norms construct [10]. Youth’s perception on values regarding sex and peers’ sexual activity determines their own sexual initiation [32, 33].

The fifth item: “*For me, to have sex is easy*” was removed, due to its low factor loading. The perceived self-efficacy item should reflect the actual control of the behaviour [21] and this may not be present in our sample. To youths, having sex may not be seen as something that they were in total control of, as premarital sex is not an accepted practice among the Malaysian population. Assessing risk-conducive circumstances may occur during their decision making [11]. At 19 years old, many of the youths had just begun college life and they stayed either in a college hostel or with their parents. The living environment may be perceived as non-conducive for sexual activity with the risk of discovery by others being a likely presence. Thus, performing sex may not be perceived as easy.

From the confirmatory factor analysis, the *p*-value of the chi-square test was <0.05, but as the chi-square test is influenced by sample size, it should not be the single index to determine model fitness [24]. Other indices that include *χ* ^2^/df, CFI, TLI and RMSEA met the recommended criteria of *χ* ^2^/df of less than 5, CFI and TLI above 0.9 and RMSEA at 0.08. These indices are sufficient to provide evidence of model fitness [29]. One may consider dropping the sexual intention item: “*I would have sex now if I could find a partner who would do it with me”*, that showed a low factor loading, in order to improve the model fitness, especially the RMSEA. Nonetheless, the authors decided not to modify the measurement model merely to improve the model fitness. This was because we felt the word “*now*” in the item was intended to reflect youths’ intention to perform sexual activity during the time of the survey. Reducing an item may improve the model fitness but this may not necessarily be the best practice [29].

Confirmatory factor analysis should be evaluated in parallel with conceptual theory, and strict adherence to the recommended cut-off may lead to type 1 error, rejecting an acceptable model [30]. Based on these and with the acceptable indices of *χ* ^2^/df, CFI, TLI and RMSEA, the YSI-Q 20 items were concluded to have satisfactory construct validity. This is further supported by the evidence of a good convergent validity in which the factor loadings were more than 0.5, average variance extracted (AVE) were above 0.5 and composite reliability (CR) were above 0.7. The discriminant validity strengthened the validity of the measurement model. Each construct of the YSI-Q was unique and were not highly correlated with other constructs. The inter-correlation between constructs was less than 0.7 and less than the square root of the AVE. The Cronbach’s alpha that measures internal consistency of each construct was more than 0.8, supporting the uni-dimensionality of each item to its respective construct [34].

This study represents an initial effort to improve our understanding of the sexual intention of youths in a conservative country like Malaysia. The cognitive components in the YSI-Q that include attitude, perception of social norms, perception of self-efficacy and sexual intention have not been extensively studied among Malaysian youths. Thus, the development and validation of the YSI-Q and the availability of this short and simple tool is timely. Hopefully, this will allow us to design culturally oriented primary intervention programs targeting youths at the intentional stage. The YSI-Q is to be used by any other population that do not allow premarital sex among their youths such as those from Arab and Asian countries. The final 20 items of the YSI-Q and instruction on how to use is available in Additional file 1.

Several limitations exist in this study and our set of questions. The Theory of Planned Behaviour (TPB) was designed to predict behaviour over which a person has control of [11], and the YSI-Q was based on this. Sexual activity is assumed to be an act that is within the control of a person and intention is the single most important determinant of sexual activity. In reality, partner’s coercion or sudden sexual arousal and desire may also influence sexual intention and subsequent sexual activity. However, these factors were not included in the YSI-Q. In addition, the use of the four-point Likert scale may not be as robust as a 7- or 10-point Likert scale. Nevertheless, as the sample of our study involved college students with different levels of academic achievement, the use of a simple scale is easier for respondents and this sample yielded a good response rate. As the YSI-Q was tested on groups of college students in Malaysia, the performance of this questionnaire on youths who are out of formal education or not in college is not known. Similarly, the YSI-Q may be more suitable for use in a conservative population such as Malaysian youths rather than in a population where safe sexual practices among youths are accepted. The YSI-Q is open to be tested in different populations and this may include youths from western and non-western countries, youths in the community either employed or non-employed and even youths of a younger age group.

## Conclusion

This study described the development and validation of the Youth Sexual Intention Questionnaire (YSI-Q) to be used among Malaysian youths. The final YSI-Q contained 20 items with five items for sexual intention, five items for attitude, six items for social norms and four items for self-efficacy. The YSI-Q showed acceptable psychometric properties, and has good internal reliability and convergent and discriminant validity. It is hoped that with the availability of this questionnaire, other researchers will further validate and perhaps use this questionnaire in their research. This consequently may enrich our understanding of sexual activity among our youths, especially those from countries where premarital sex is prohibited.

## Additional file

Additional file 1: Youth Sexual Intention Questionnaire (YSI-Q). This file contains a brief description about YSI-Q, instruction on how to use and the final 20 items of the YSI-Q. (PDF 728 kb)

## Abbreviations

AMOS: Analysis of moment structure
AVE: Average variance extracted
CFA: Confirmatory factor analysis
CFI: Comparative fit index
CR: Composite reliability
EFA: Exploratory factor analysis
KMO: Kaiser-Meyer-Olkin
RMSEA: Root mean square error of approximation
TLI: Tucker Lewis Index
TPB: Theory of planned behaviour
YSI-Q: Youth sexual intention questionnaire
