# Supplementary material for: Questionnaire development and validity to measure sexual intention among youth in Malaysia
Source: BMC Public Health. 2017 Feb 2;17:157. doi: 10.1186/s12889-016-3949-1 (PMC5289019; doi:10.1186/s12889-016-3949-1)
Supplement: Additional file 1: — Youth Sexual Intention Questionnaire (YSI-Q). This file contains a brief description about YSI-Q, instruction on how to use and the final 20 items of the YSI-Q. (PDF 728 kb) [file 12889_2016_3949_MOESM1_ESM.pdf]

Supplementary 1

UNIVERSITI KEBANGSAAN MALAYSIA
National University of Malaysia

YOUTH SEXUAL INTENTION QUESTIONNAIRE (YSI-Q)

Authors:
Noor Azimah Muhammad, Khadijah Shamsuddin, Rahmah Mohd. Amin and Khairani Omar

INTRODUCTION

The Youth Sexual Intention Questionnaire (YSI-Q) is a 20 item self-administered questionnaire and was constructed based on the Theory of Planned Behaviour. It is designed to measure current sexual intention of both male and female unmarried youths. YSI-Q is scored based on the responses on a 4-point Likert scale from 1 (strongly disagree) to 4 (strongly agree). A higher total score of item 1 to 5 indicates a higher intention to have sex, a higher total score of item 6 to 10 indicates a higher permissive attitude towards premarital sex, a higher total score of item 11 to 16 indicates a higher perception of social norms on premarital sex and a higher total score of item 17 to 20 indicates a higher perception of self-efficacy on performing sexual activity. The Malay version of the YSI-Q is available upon request.

YOUTH SEXUAL INTENTION QUESTIONNAIRE (YSI-Q)

The following statements are about sexual activities among unmarried youths. Please read each statement carefully and circle a number 1, 2, 3, or 4 which indicates how much you agree or disagree with the statement. There is no right or wrong answer. Please give your honest response for all the items.

**OPTIONS:**	**Strongly disagree**<br>1	**Disagree**<br>2	**Agree**<br>3	**Strongly agree**<br>4

1.	I expect to have sex with my partner	1 2 3 4
2.	I want to have sex with my partner	1 2 3 4
3.	I intend to have sex with my partner	1 2 3 4
4.	I would like to have sex to see what it is like.	1 2 3 4
5.	I would have sex now if I could find a partner who would do it with me	1 2 3 4
6.	I believe a sexual encounter that lasts only once is all right.	1 2 3 4
7.	I believe youths who have never been involved in sexual intercourse before marriage are old-fashioned.	1 2 3 4
8.	Youths should have sex before their marriage to see whether they are suited to each other.	1 2 3 4
9.	Youths can have sex provided they use methods to stop pregnancy.	1 2 3 4
10.	Youths can have sex if they are unable to control their sexual desire.	1 2 3 4
11.	Most of my friends are practicing sex before marriage.	1 2 3 4
12.	Most of my friends think it is mature to practice sex at my age.	1 2 3 4
13.	Most of my friends think female youths do not have to maintain their virginity.	1 2 3 4
14.	Most of my friends think male youths are allowed to practice sex before marriage.	1 2 3 4
15.	Most of my friends think that you can have sex before marriage if you are in love.	1 2 3 4
16.	Most of my friends think youths who have never been involved in sexual intercourse before marriage are old-fashioned.	1 2 3 4
17.	I know when I can have sex.	1 2 3 4
18.	I know where I can have sex.	1 2 3 4
19.	I can decide on my sexual activity.	1 2 3 4
20.	Whether I have sex or not is entirely up to me.	1 2 3 4

Thank you for your participation
